# Loading of anionic surfactant on eco-friendly biochar and its applications in Cr(VI) removal: adsorption, kinetics, and reusability studies

**DOI:** 10.1186/s13065-024-01363-4

**Published:** 2025-01-10

**Authors:** Azza M. Shaker, Mohamed Khedawy, Abeer A. Moneer, Nabila M. El-Mallah, Mohamed S. Ramadan

**Affiliations:** 1 Chemistry Department, Faculty of Science, Alexandria University, Alexandria, Egypt; 2 Marine Pollution Department, Environmental Division, National Institute of Oceanography and Fisheries, NIOF, Alexandria, Egypt

**Keywords:** Cr(VI) removal, Modified biochar, Kinetic studies, Adsorption isotherms, Thermodynamic parameters

## Abstract

Surfactant-modified biochar is a viable adsorbent for eliminating Cr(VI) from synthetic wastewater. The biochar obtained from the *zea mays* plant (BC) was tailored with sodium dodecyl sulfate (SDS) as an anionic surfactant forming SDS-BC adsorbent. Different controlling conditions have been evaluated including pH of the solution, biomass concentration, primary Cr(VI) concentration, time of adsorption, and temperature. Under the best controlling circumstances, the percentage of removal has attained 99%. The pseudo-second-order kinetic model best described the removal process, according to the kinetic data, while the Temkin model, one of the applicable adsorption isotherm models, well expressed the adsorption process. The thermodynamic parameters were computed, which disclosed the spontaneity and exothermic character of the Cr(VI) elimination. According to the regeneration cycles, SDS-BC was cost-effective and had a good removal capability.

**Graphical Abstract:**

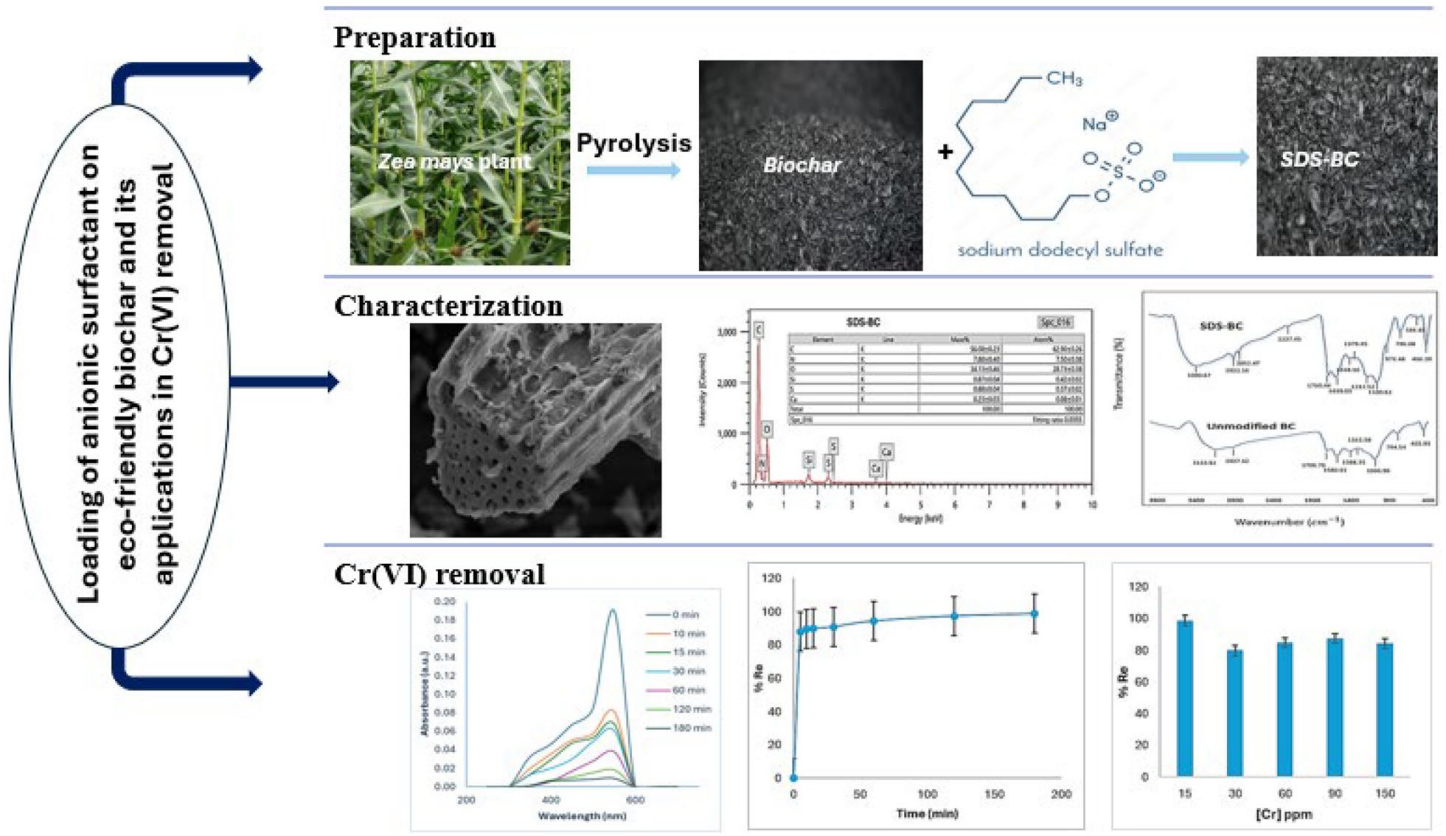

## Introduction

Water pollution is a major environmental issue that results from direct and indirect contamination of water bodies. Water pollution can lead to a reduction in water quality and constitute a serious risk to humans and the ecosystem. Aquatic habitat deterioration, biodiversity loss, and food chain disruption can result from contaminants such as heavy metals, pesticides, pharmaceuticals, and pathogens [1].

Heavy metals like Chromium, lead, mercury, and arsenic are important for industrial and agricultural applications. However, they pose significant dangers associated with their toxicity and environmental persistence. They can settle in sediments and aquatic animals which may cause bioaccumulation in the food chain by entering the water bodies from industrial discharges, mining operations, or agricultural runoff. In addition to killing and disturbing aquatic life, this pollution puts human health at serious risk when it comes to neurological conditions, renal damage, and other types of cancer. It also contaminates water and seafood consumers [2, 3].

A common heavy metal that exists in wastewater is chromium. Cr(III) and Cr(VI) are two oxidation states in which chromium is commonly observed. Still, Cr(VI) is nearly a thousand times extra poisonous than Cr(III) [4]. Given its extreme toxicity and carcinogenic properties, Cr(VI) pollution in aquatic environments is especially concerning. The industrial processes of electroplating, tanning leather, and textile manufacture are common sources of this pollution [5]. Due to its extraordinary solubility and ease of penetration through biological membranes, Cr(VI) can cause extensive contamination in water. Drinking water or skin contact exposure to Cr(VI) can result in serious health concerns like skin ulcers, lung and gastrointestinal malignancies, and respiratory disorders [6]. To safeguard human health and environmental quality, strict pollution control measures and efficient remediation procedures are needed, given the persistence of Cr(VI) in the surroundings and its detrimental consequences [7].

Numerous advanced technologies have been developed for treating wastewater containing Cr(VI), including chemical precipitation, membrane filtration, ion exchange, electrocoagulation, and adsorption [8–12]. Among these, adsorption is a widely preferred technique due to its simplicity, efficiency, and cost-effectiveness. Unlike more complex methods that often require high operational costs or extensive energy inputs, adsorption offers a scalable and practical solution, particularly for regions with limited resources.

Biochar, a carbon-rich material derived from the pyrolysis of organic biomass, has emerged as a promising adsorbent for the removal of various heavy metals, including Cr(VI) [13]. Its large surface area, porous structure, and abundance of functional groups contribute significantly to its adsorption capabilities [14, 15]. The use of biochar promotes sustainability by utilizing agricultural waste through transforming organic residues into high-value adsorbents, reducing waste disposal issues, and supporting circular economy practices.

Extensive research has explored the modification of biochar using surfactants and other chemical agents. For instance, sodium dodecyl sulfate (SDS), an anionic surfactant, and tetradecyl trimethyl ammonium bromide (TTAB), a cationic surfactant, have been shown to enhance biochar’s adsorption performance by improving surfactant functionality and altering its pore structure [16]. Similarly, surfactants like sodium dodecyl benzene sulfonate (SDBS), cetyltrimethylammonium bromide (CTAB), Gemini surfactant, nonionic surfactants, and combinations of double surfactant-modified systems have demonstrated significant improvements in adsorption efficiency. These modifications can increase surface charge, enhance hydrophobicity, and optimize the adsorbent's structural properties, thereby facilitating Cr(VI) removal from aqueous solutions [17–21].

Despite these advancements, several challenges remain. Many previous studies have focused on laboratory-scale experiments with limited exploration of real-world applications or scaling up these techniques for industrial use. Additionally, while surfactant modifications improve adsorption efficiency, the long-term stability of modified biochars and their regeneration potential require further investigation. These gaps emphasize the need for systematic studies that evaluate both the immediate and sustainable performance of modified biochars under diverse environmental conditions.

The safe disposal or regeneration of biosorbents after Cr(VI) removal also presents a critical challenge. Secondary environmental contamination must be avoided, necessitating reliable disposal methods such as thermal treatment, chemical stabilization, and controlled landfill disposal [22, 23]. Emerging strategies like regeneration and composting, where feasible, provide more sustainable options but require further validation. Addressing these issues holistically can significantly enhance the environmental and economic viability of biochar-based adsorption systems.

The study highlights the environmental and economic benefits of biochar as a green, multifunctional material by combining waste management with water purification. *Zea mays* biochar was loaded with SDS, an anionic surfactant, to enhance its carbon content and functional groups, improving its efficiency for Cr(VI) ion uptake. The preparation and adsorption process are detailed, followed by the characterization of biochar before and after modification. Batch adsorption experiments were conducted to identify optimum conditions, and the adsorption behavior was analyzed using kinetic models and isotherms.

## Experimental

### Materials

Cr(VI) ions were obtained from potassium dichromate (K_2_Cr_2_O_7_) salt (99%, universal fine chemicals). Sulfuric acid (H_2_SO_4_) and sodium hydroxide (NaOH)) were used in the solution pH alteration. 1,5-diphenylcarbazide (99.0% Sigma Aldrich) for complexation with Cr ions. Sodium dodecyl sulfate surfactant (SDS) (99%) for the modification of BC. All chemicals are obtained commercially and used as they are without further purification.

### Instrumentation

Table 1 shows the instruments used throughout the study and their characteristics.

**Table 1:** The characteristics of the instruments used for chromium removal

Instrument	Characteristics
T80 UV/Vis spectrophotometer	Deuterium lamp for UV region (190–350 nm), halogen lamp for the VIS/NIR region (340–1100 nm)
Fourier Transform Infrared (FT-IR)	BRUKER Tensor 37 with KBr pellets (FT-IR) in the range of 400–3900 cm^−1^
Scanning Electron Microscope (SEM)	JEOL-JSM-IT200 microscope
Energy-dispersive X-ray spectroscopy (EDX)	JEOL-JSM-IT200 microscope
pH meter	Adwa instruments calibrated with standard buffer

### Adsorbent preparation

The biochar was prepared from the leaves of the corn plant [*zea mays*] which was obtained locally from the farms in Beheira Governorate and air dried at room temperature. The biomass was then crushed, put in capped crucibles, and subjected to slow pyrolysis under limited oxygen conditions at 300 °C for 5 h. The biochar was then activated by 1 M H_2_SO_4_ for 12 h at 70 °C then filtered and washed using purified water several times then dehydrated at 75 °C for 4 h. The resultant biochar was then modified by sodium dodecyl sulfate (SDS) surfactant.

10 g of the produced biochar was incorporated into a 100 ml of the SDS solution of 0.008 M concentration which matches the critical micelle concentration (CMC) of that surfactant. The mixture was agitated for 12 h, followed by filtering and oven drying at 75 °C for 4 h and the modified biochar (SDS-BC) was stored in a glass container protected from air.

### Batch adsorption experiments

An investigation of Cr(VI) adsorption on the SDS-BC was conducted using a batch approach. Parameters like pH, mass of adsorbent, concentration of adsorbate, contact time, and temperature were studied. One gram of K_2_Cr_2_O_7_ was dissolved in one liter of distilled water to prepare Cr(VI) stock solution. Various concentrations were then obtained through dilution from this stock solution. Prior to each experiment, the solutions’ pH was changed using 0.5 molar NaOH and 0.5 molar H_2_SO_4_.The vials were situated in a thermostat shaker and agitated at 300 rpm for 180 min. The adsorbent dose varied between 0.05 g and 0.4 g. The preliminary Cr(VI) concentration varied from 15 to 150 ppm to study the adsorption isotherms. The experiment was done at different temperatures from 20 to 60 °C and the thermodynamic parameters were investigated. To investigate the kinetic models that most closely matched the adsorption process, the impact of the time of contact was assessed from 5 to 180 min. The Cr(VI) concentration was followed spectrophotometrically via the complexation with 1,5-diphenylcarbazide in acidic medium at 542 nm wavelength. The quantity of adsorbed Chromium (q) (mg/g) and the removal percentage (%Re) were evaluated by Eqs. (1, 2).

1 $$q=\frac{\left({C}_{o}-{C}_{t}\right)x V}{m}$$

2 $$\%Re=\frac{({C}_{o}-{C}_{t})}{{C}_{o}}x100$$

where the ${C}_{o}$ is the preliminary chromium concentration (mg/L), ${C}_{t}$ stands for the chromium concentration at equilibrium adsorption time (ppm), $V$ stands for the volume of chromium aqueous solution (L) and m denotes the mass of SDS-BC (g).

## Results and discussion

### SDS-BC characterization

#### SEM

**Fig. 1:**
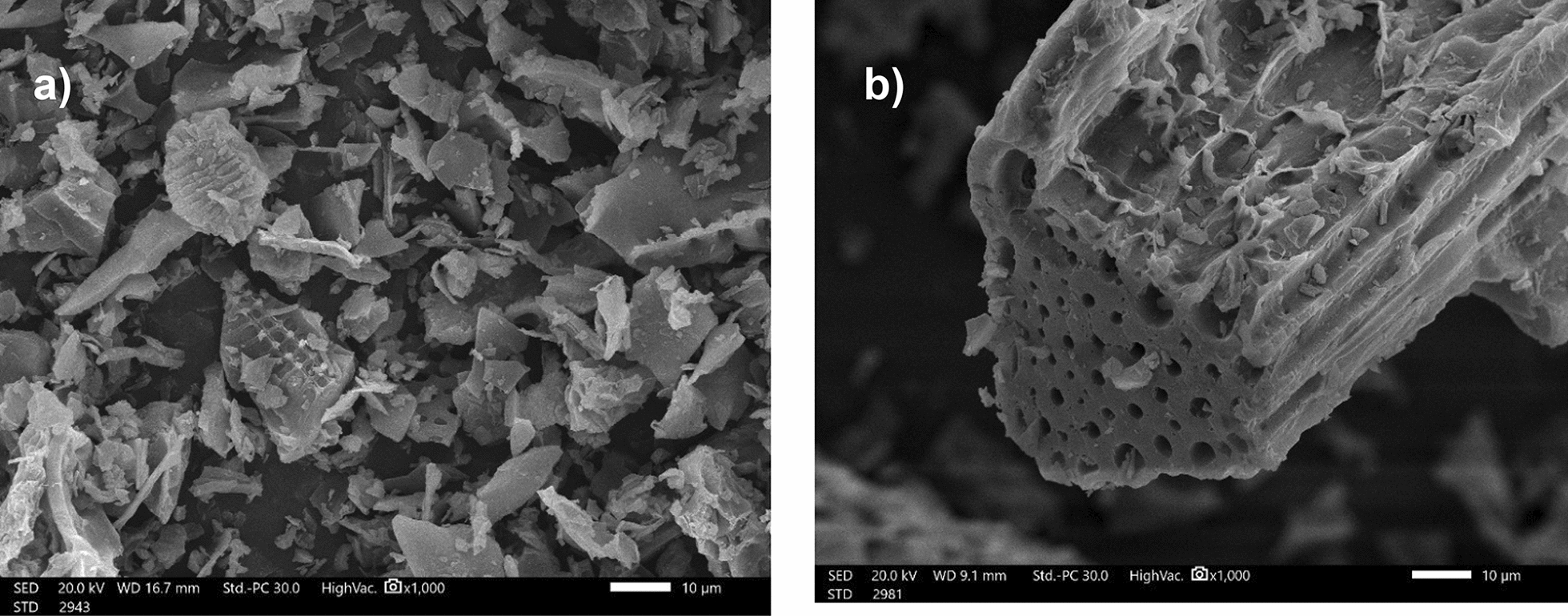
SEM analysis. **a** BC, **b** SDS-BC

The SEM analysis is an important instrument for visualizing the morphology of the surface of the biochar. Figure 1a represents the SEM image for the unmodified biochar (BC). From the figure, there are so many pores observed in the biochar however, these pores are small in size and scattered. In the SDS-BC Fig. 1b, more pores emerged on the surface after the modification with SDS surfactant. The modification improved the amount and the volume of the holes which reveal more homogeneous adsorption sites for the binding of metals.

#### FT-IR

**Fig. 2:**
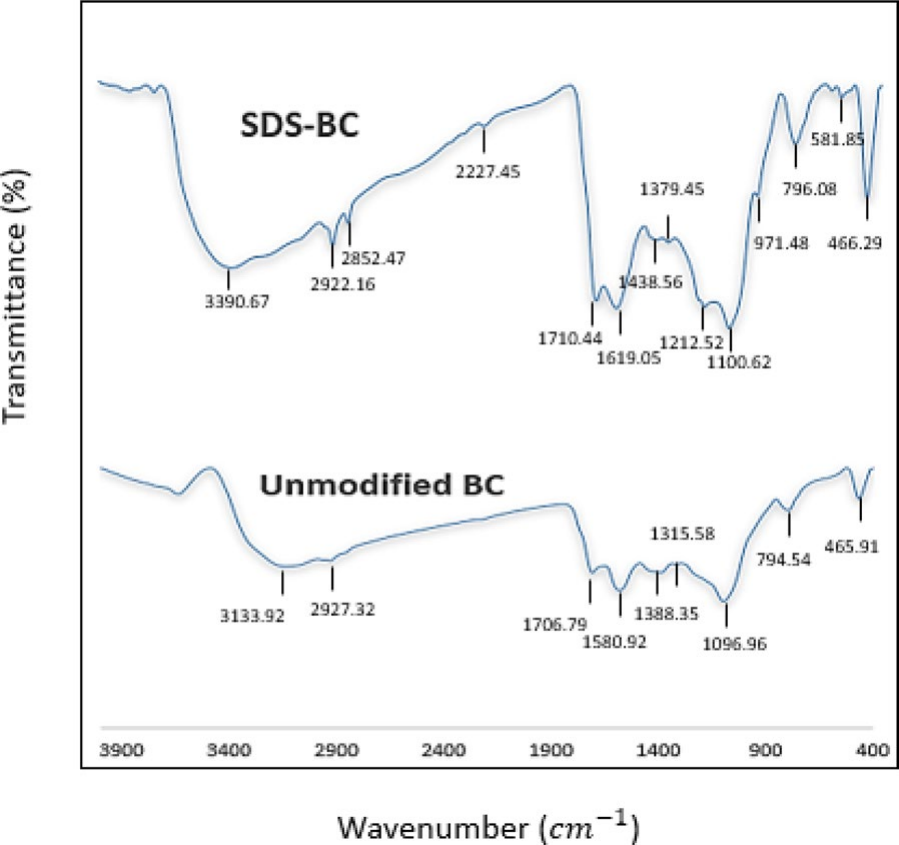
BC and SDS-BC FT-IR spectra

Figure 2 illustrates the FT-IR spectra of the BC and the SDS-BC. These peaks reveal the complex chemical composition of BC and SDS-BC, highlighting the presence of aromatic structures, various oxygen-containing functional groups, aliphatic hydrocarbons, and mineral impurities. Table 2 illustrates the identifications of FTIR peaks for both BC and SDS-BC [24].

**Table 2:** Adsorbent FT-IR peaks identifications

Peaks of BC (cm^−1^)	Identification of the peaks	Peaks of SDS-BC
3133.9	O–H stretching vibrations (hydroxyl groups)	3350.6
2927.3	C–H stretching in aliphatic methylene groups	2922.2 and 2852.5
–	S–H stretching vibrations	2227.4
1706.8	C = O stretching in carbonyl groups	1710
1580.9	C = C stretching in aromatic rings	1619
1388.3	C–H bending in aliphatics or carboxylate anions	1438.5
-	C–H bending in aliphatics or -SO_3_ stretching	1379.4
1315.6	C–H bending in aromatics or C–O stretching in carboxylic acids, esters, or ethers	–
1096.9	C–O stretching in alcohols, carboxylic acids, esters, or ethers or S = O stretching in SDS-BC	1100.6 and 1212.5
-	C–H out-of-plane bending in alkenes or S = O stretching	971.5
794.5	Out-of-plane C–H bending in aromatics	796 and 851.8
465.9	Si–O bending vibrations	466.3

#### EDX

**Fig. 3:**
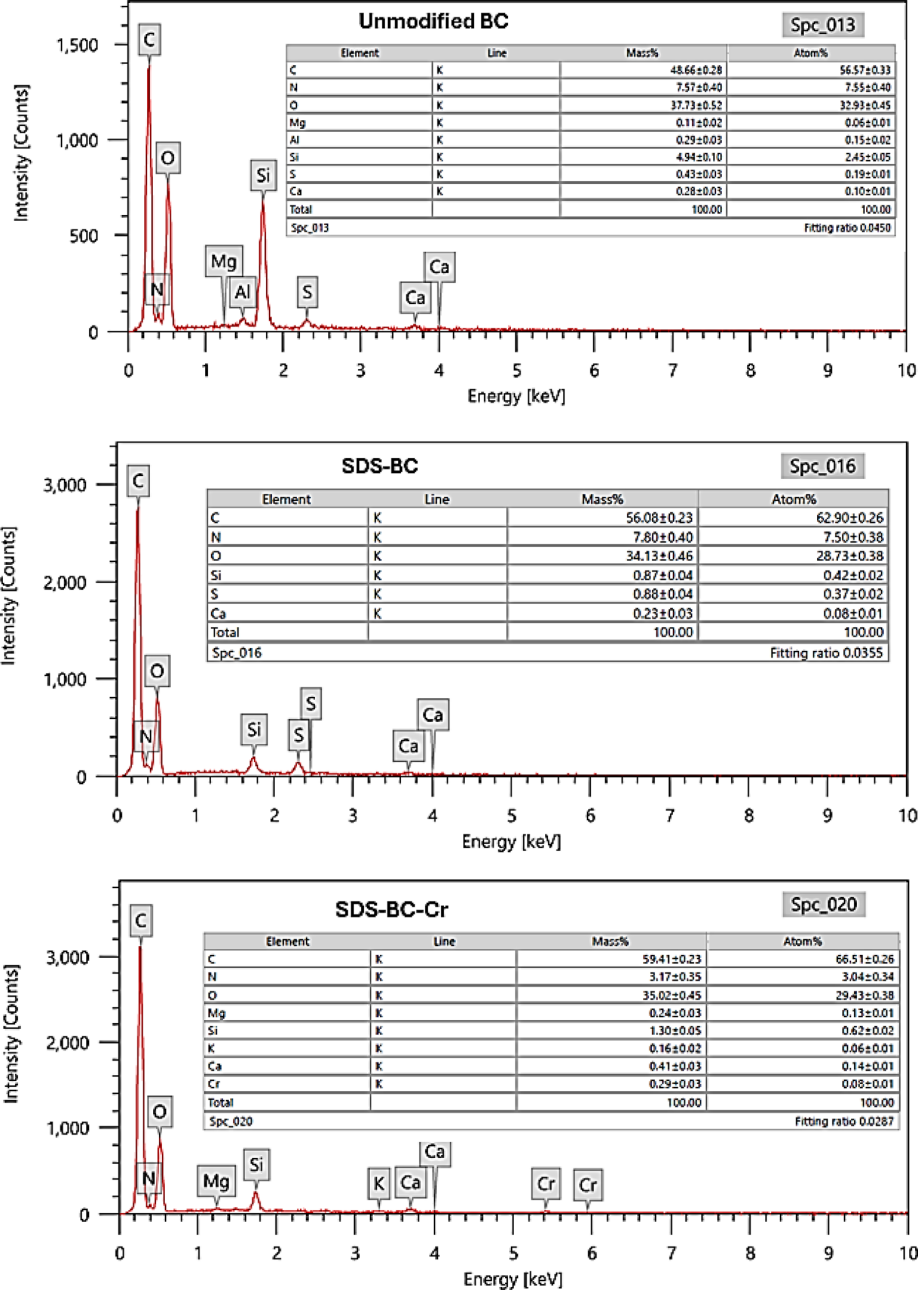
EDX of BC, SDS-BC, and SDS-BC-Cr

Figure 3 illustrates the EDX spectra of the BC, SDS-BC, and SDS-BC-Cr obtained after the treatment process. As depicted in the figure, the biochar exhibits a high carbon content, apparent from the prominent peak corresponding to the C element. The presence of oxygen, indicated by the peak corresponding to the O element, suggests the existence of various oxygen-containing functional groups that influence the BC’s reactivity and stability. The presence of silicon (Si) contributes to the structural stability of biochar. Some other trace elements may coexist within the biochar in very small amount as represented in the figure.

In SDS-BC sample, the carbon chain of the SDS surfactant contributed to an increase in the carbon content, as evidenced by a higher carbon peak compared to the BC sample. Additionally, the presence of sulfur in the SDS-BC sample confirms the successful modification of the biochar with SDS. After the treatment process, the modified biochar was able to eliminate the Cr (VI) ions from wastewater which can be confirmed by the presence of a Cr peak in the EDX spectrum.

### Parameters optimization

#### pH’s impact

**Fig. 4:**
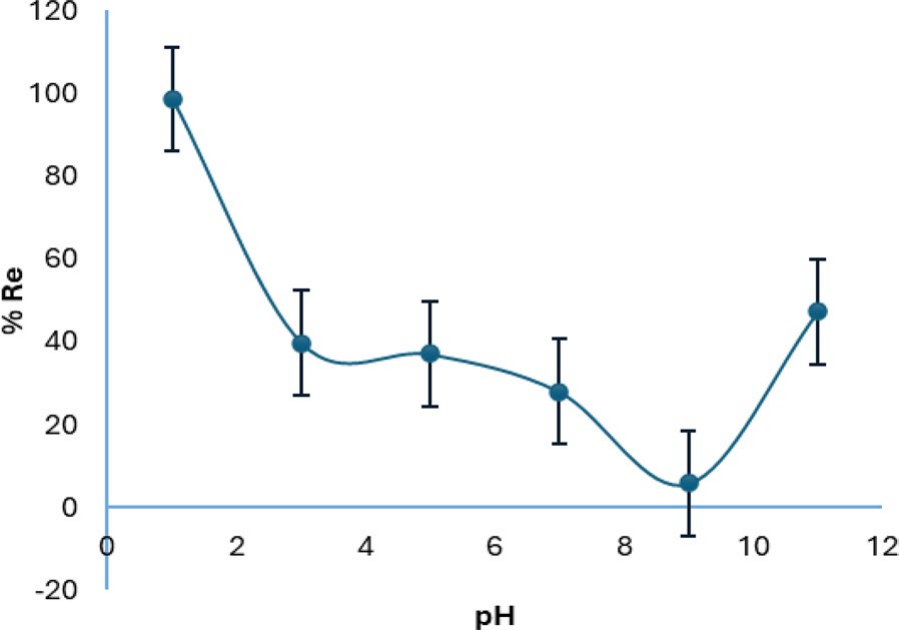
pH influence on the removal of Cr(VI)

**Fig. 5:**
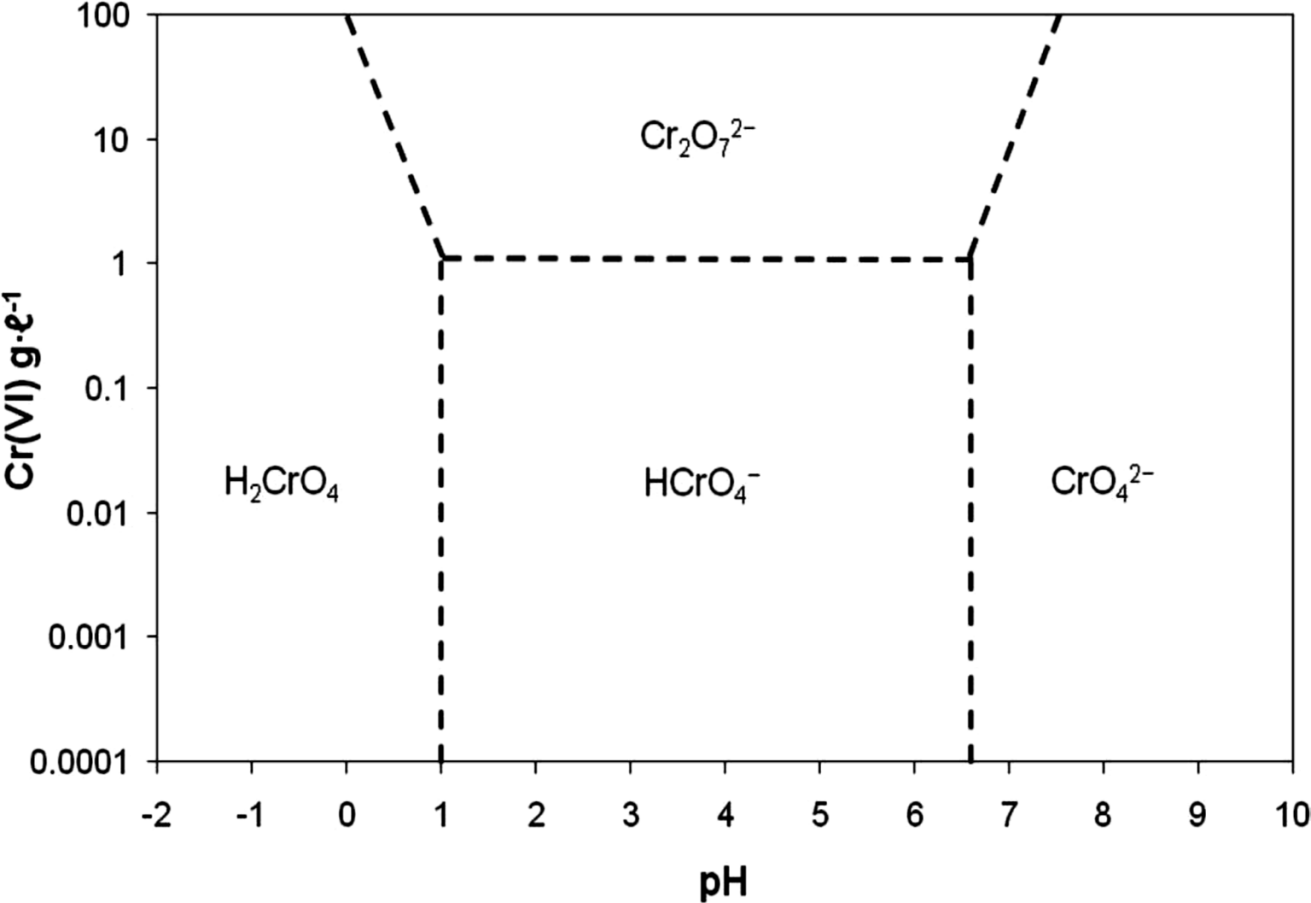
Distribution of Cr species in aqueous solution as a function of pH

Figure 4 shows the impact of pH of the solution on the efficacy of Cr(VI) removal. The maximum elimination was achieved in acidic medium. However, the removal decreased at higher pH conditions. At lower pH values, the most dominant species of Cr is the HCrO_4_^−^, which exists in pH from 1 to 6 consistent with Fig. 5 which illustrates the relative distribution of Cr(VI) [25], which brings about an electrostatic contact between the positively charged adsorbent and the current species. At low pH, the SDS-BC surface was protonated, and the surface obtained a positive charge under acidic conditions. The positively charged adsorbent attracted the negatively charged chromium species present in the solution which resulted in a very high efficacy of removal of Cr(VI) at pH = 1 (%Re = 98.65%) compared to the other pH values.

#### Biomass concentration effect

**Fig. 6:**
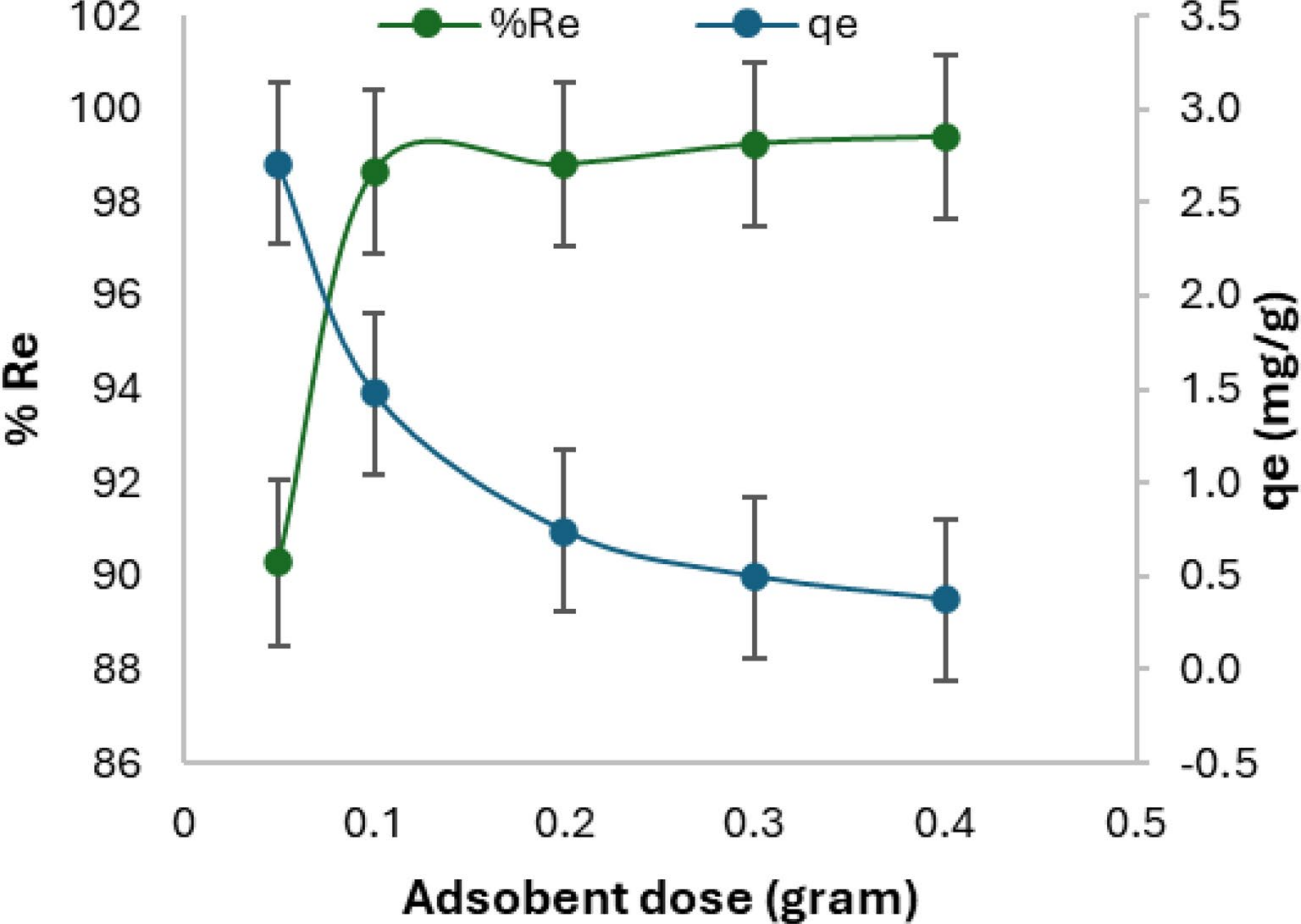
Adsorbent dosage effect on Cr(VI) removal

Figure 6 demonstrates the impact of the adsorbent dose on Cr(VI) removal. Inspecting the figure, with increasing the dosage of adsorbent the removal increased from 90% to 99.4%. The removal has increased due to the adsorbent’s wide surface area and the availability of sites for the adsorption process. The high porosity and higher surface area of SDS-BC caused a higher elimination of Cr(VI). In contrast, by increasing the dosage of SDS-BC, the amount of active sites increased and the ratio of chromium ions to the number of sites decreased causing a decrease in the adsorption capacity (q_e_).

#### Cr (VI) primary concentration effect

**Fig. 7:**
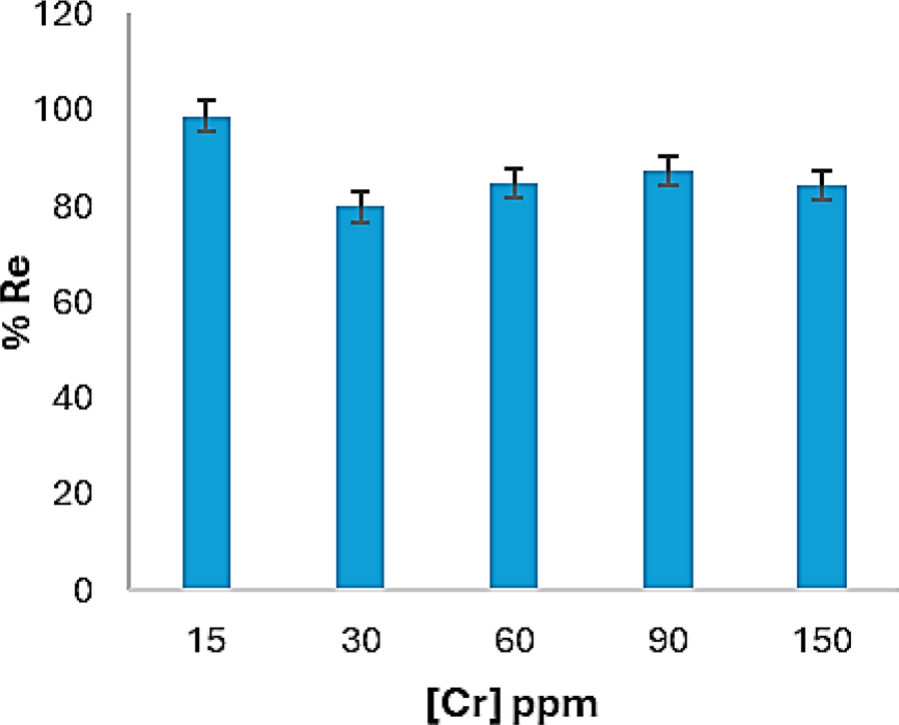
Cr(VI) concentration influence on the efficacy of removal

The study examined the impact of Cr(VI) concentration, utilizing concentration ranges from 15 to 150 ppm, as illustrated in Fig. 7. According to the figure, with increasing the concentration of Cr(VI) the removal efficiency diminished, and the best removal efficacy was observed with a concentration of 15 mg/L, 0.1 g of the biomass, and at pH 1 which gave removal of 98.6%. The reduction in removal could be assigned to the saturation of the adsorbent active sites with Cr(VI) where further increase in Cr(VI) concentration leads to lowering in the removal efficiency and no adsorption would take place.

#### Impact of duration of contact

**Fig. 8:**
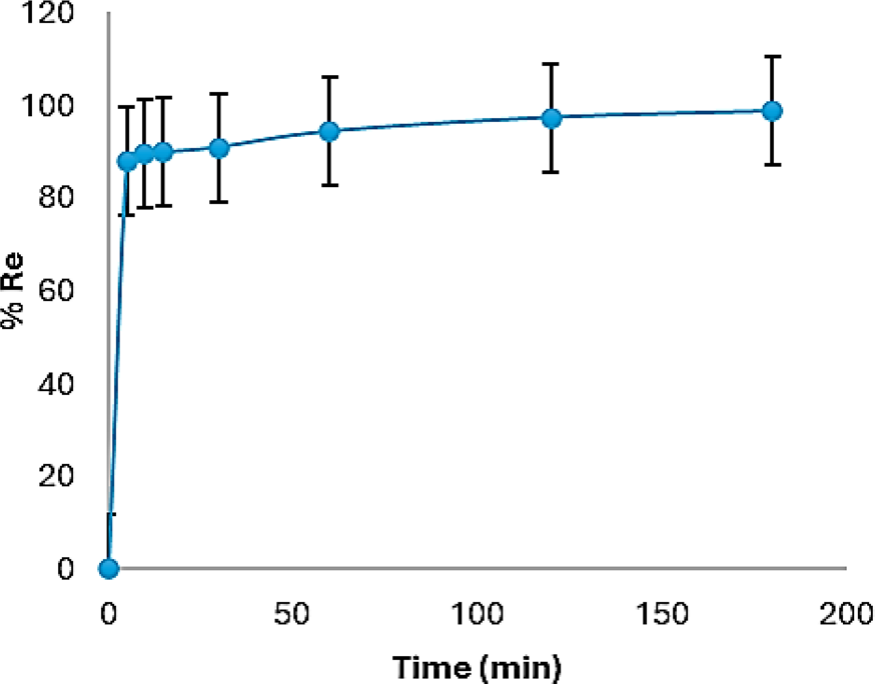
Impact of duration of contact on the efficacy of removal of Cr(VI)

The rate of a chemical reaction can be determined through the duration of contact between the adsorbent and the adsorbate. Figure 8 explains the influence of the duration of contact on the efficacy of Cr(VI) removal. From the figure, the modified biochar showed a removal of 87.9% in the first 5 min. Then, the removal slowly increased with increasing the contact time to 180 min where the removal reached 98.6%. This observation could be attributed to the existence of adequate adsorption sites on the SDS-BC which led to a high adsorption rate in the first 5 min afterward, the adsorption sites declined, and the adsorption rate reduced with increasing the duration of contact. At equilibrium, the adsorption process reached saturation and the adsorption sites decreased.

**Fig. 9:**
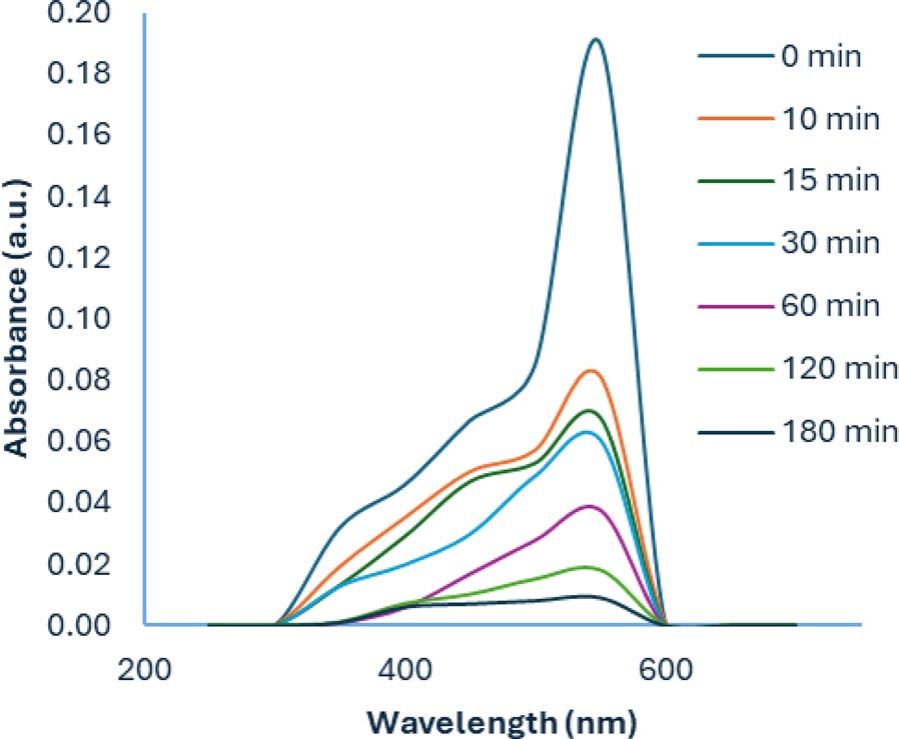
UV–Vis spectra of Cr(VI) removal by SDS-BC with time

Figure 9. Represents the variation of the UV–Vis absorbance spectrum of Cr(VI) removal by SDS-BC with time. The complex formed with diphenyl carbazide gave a peak with a maximum wavelength of 542 nm. As can be depicted from the figure, there is a decrease in the absorbance of Cr(VI) ions with time resulting from the reduction in Cr(VI) concentration. This signifies the successful removal of Cr(VI) by SDS-BC.

#### Temperature effect

**Fig. 10:**
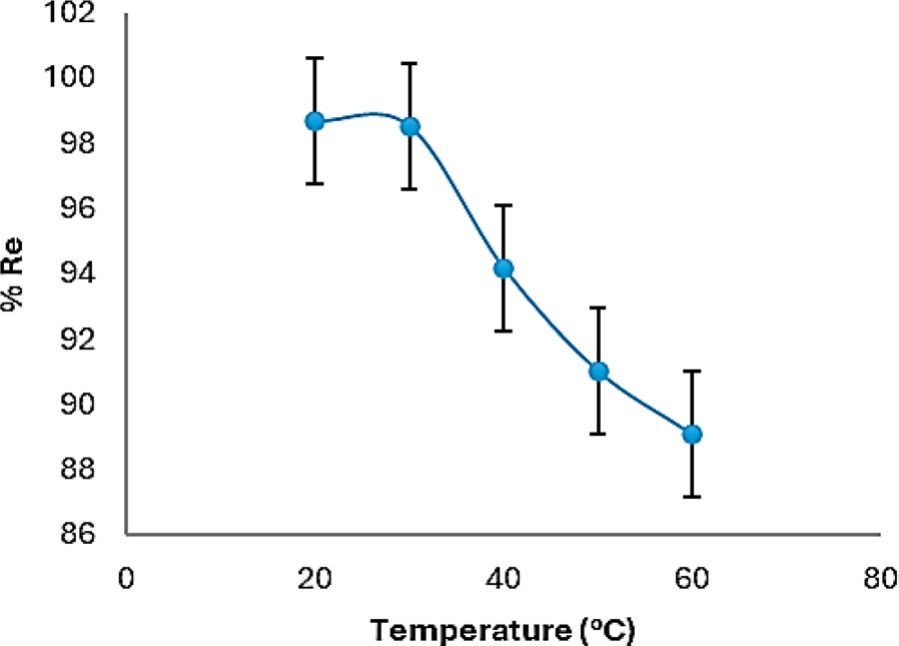
Temperature’s impact on the efficacy of removal of Cr(VI)

One important factor that significantly influences the adsorption capacity is temperature. The effect of temperature on the elimination of Cr(VI) is displayed in Fig. 10. The % Re was almost the same at 20 and 30 °C where it was 98.6 and 98.5% respectively. However, upon increasing the temperature the removal started to decline which could be due to the higher thermal agitation of the solution. The adsorbent and adsorbate collide by means of this agitation, which is detrimental to the SDS-BC’s ability to adsorb Cr(VI), therefore 20 °C was the optimum temperature. The thermodynamic parameters were evaluated and tabulated in Table 3. From the table, the best temperature is at 20 °C where the value of standard free energy change (**∆G**^**o**^) is highly negative indicating a spontaneous process of adsorption than its value at higher temperatures. The standard enthalpy change’s (**∆H**^**o**^) negative value signifies an exothermic adsorption process, and the standard entropy change’s (**∆S**^**o**^) negative value reveals that the adsorption process was ordered. The thermodynamic parameters were calculated according to shaker et al. [26].

**Table 3:** Thermodynamic parameters for Cr(VI) removal

Temperature	∆G^o^ (kJ/mol)	∆H^o^ (kJ/mol)	∆S^o^ (J/mol. K)
293	− 4.85356	− 50.9191	− 156.079
303	− 4.74998		
313	− 1.24798		
323	− 0.03998		
333	0.561475		

### Kinetic studies

Table 4 shows the pseudo-first order, pseudo-second order, and Elovich models that were applied to the experimental data of Cr(VI) removal by SDS-BC. The pseudo-second-order kinetic model appeared to be the most applicable to describe the adsorption procedure, as the table indicates, as its correlation coefficient (R^2^) was greater than that of the other two models. Furthermore, the value of q_e_ computed using the pseudo-second-order model was nearly identical to the experimental value acquired from the experiment; hence the value of q_e_ and R^2^ indicated that the pseudo-second-order model best defined the removal process.

**Table 4:** Equations and parameters of different kinetic models for Cr(VI) removal

Kinetic model	Parameters	Value	Equation	References
	q_e_ (experimental)	1.479		
Pseudo first order	K_1_	0.017733	$\mathit{log}\left({q}_{e}-{q}_{t}\right)=\mathit{log}\left({q}_{e}\right)-\frac{{k}_{1}t}{2.303}$ ${q}_{e}$ (mg/g): equilibrium adsorption capacity q_t_ (mg/g): quantity of metal adsorbed at time t *k*_*1*_ (min^−1^): pseudo-first-order rate constant	[26]
	R^2^	0.9905		
	q_e_	0.177		
Pseudo second order	K_2_	0.492821	$\frac{t}{{q}_{t}}= \frac{1}{{k}_{2}{q}_{e}^{2}}+\frac{t}{{q}_{e}}$ ${k}_{2}$(g mg^–1^ min^–1^): pseudo-second-order rate constant	[26]
	R^2^	0.9997		
	q_e_	1.463		
Elovich	$\beta$	21.786	${q}_{t}=\left(\frac{1}{\beta }\right)\text{ln}\left(\alpha \beta \right)+\left(\frac{1}{\beta }\right)\text{ln}t$ α (mg g^−1^ min^−1^): initial adsorption rate β (mg g^−1^): is connected to the degree of surface covering and the chemisorption activation energy	[27]
	$\alpha$	2.0785 × 10^10^		
	R^2^	0.953		

### Adsorption isotherms

**Fig. 11:**
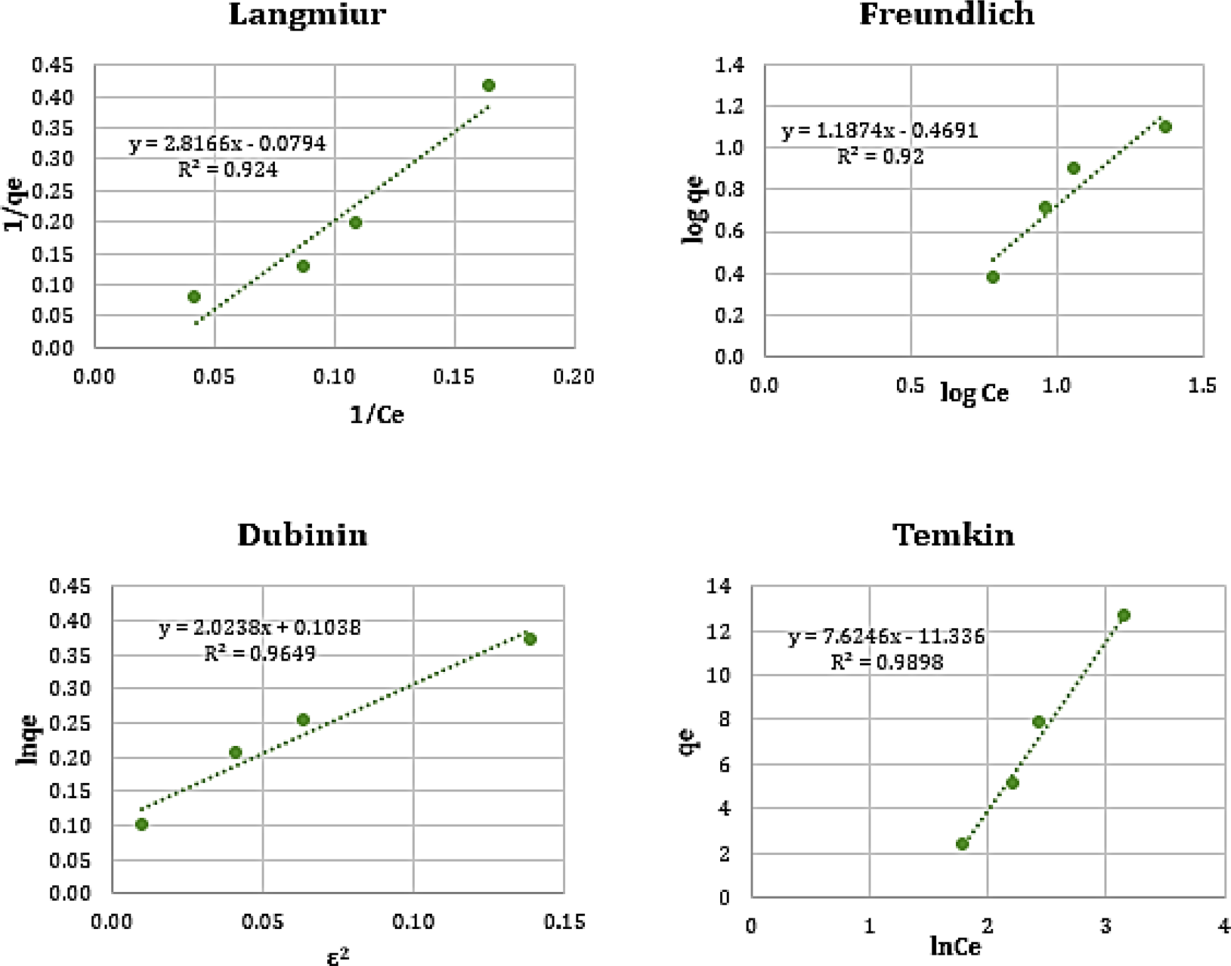
Different Adsorption isotherm plots

Adsorption isotherm is essential in interpreting how the SDS-BC will interact with the Cr(VI) metal. The adsorption model gives an idea of the capacity of adsorption and the adsorption mechanisms. Four models were examined involving Langmuir, Freundlich, Dubinin-Radushkevich (D-R), and Temkin (Fig. 11). The applicability of these models is predicated on the correlation value (R^2^) value. Table 5 shows the adsorption isotherm equations and parameters obtained. Temkin isotherm model, which postulates that adsorption reduces linearly as adsorbent surface coverage rises, was found to be a good fit for the adsorption process, indicated by Table 5 [16].

**Table 5:** Adsorption isotherm parameters and equations for Cr(VI) removal

Adsorption model	Parameters	Value	Equation	References
Langmuir	R^2^	0.924	$\frac{1}{{q}_{e}}=\left[\frac{1}{{K}_{a}{Q}_{m}}\right]\frac{1}{{C}_{e}}+\frac{1}{{Q}_{m}}$ K_a_ (L/mg): Langmuir constant Q_m_ (mg/g): maximum adsorption capacity *C*_*e*_ **(**mg/L): equilibrium concentration of adsorbate	[28, 29]
	K_a_	− 0.028		
	Q_m_	− 12.59		
Freundlich	R^2^	0.920	$\mathit{ln}{q}_{e}= \mathit{ln}{K}_{f}+\frac{1}{n}\mathit{ln}{C}_{e}$ n: Heterogeneity factor K_f:_ (mg/g) (L/mg)^1/n^ Freundlich constant	[30]
	*n*	0.842		
	K_f_	0.339		
Dubinin–Radushkevich (D–R) isotherm	R^2^	0.965	$\text{ln}{q}_{e}=\text{ ln}{q}_{m}-K\varepsilon$ ^*2*^ K: (mol^2^/J^2^) D-R constant ***ε*** Polanyi potential	[31]
	K	− 2.024		
	q_m_	1.109		
Temkin	R^2^	0.989	${q}_{e}={B}_{T}ln{A}_{T}+{B}_{T}ln{C}_{e}$ ${B}_{T}=\frac{RT}{{b}_{T}}$ B_T_ (J/mol): constant related to the heat of adsorption A_T_ (L/mg): Temkin adsorption potential	
	B_T_	7.625		
	A_T_	0.226		

## Regeneration and reusability

According to the repeated application cycles, it is essential to look at the reusability of various biosorbents, such as the SDS-BC, as they play a big role in the economy. The recycling reagent HCl was used in this study to investigate the regeneration of SDS-BC biosorbent. For the first, second, and third rounds of regeneration, respectively, the effectiveness of removal values of Cr(VI) ions by the SDS-BC correlated to 91.03, 91.33, and 91.78. Accordingly, the regenerated SDS-BC is a great environmentally friendly modified biochar that can be utilized to eliminate Cr(VI) from wastewater.

## Comparison with other studies

The adsorption capacity of Cr(VI) on SDS-BC was evaluated against various other adsorbents, as presented in Table 6. The differences in the q values across these adsorbents can be attributed to variations in their elemental composition, morphology, particle size, pore volume, and the presence of diverse functional groups.

**Table 6:** Comparison of Cr(VI) adsorption capacity on SDS-BC with other modified biochar

Biochar	pH	[Cr^6+^] (mg/L)	Contact time (min)	q (mg/g)	References
Magnetic biochar	3	30	180	25.94	[32]
Biochar modified with cationic surfactant (C_16_H_33_)N(CH_3_)_3_Br	6	100	480	52.63	[33]
Biochar modified with cationic surfactant Cetyltrimethyl Ammonium Bromide (CTAB)	2	125	120	22.3	[34]
Biochar modified with magnetite nanoparticles	4	200	180	77.35	[35]
Chitosan-modified magnetic biochars	2	200	1440	127	[36]
SDS-BC	1	150	180	12.6	Our work

## Conclusion

This work deals with the study of the feasibility of SDS-BC used in the adsorption of Cr(VI). The characterization of the modified biochar indicated that the SDS increased the adsorption sites through the increase of the hydrophilic and hydrophobic groups on biochar and increasing its surface area allowing better diffusion of Cr(VI) ions into the interior of the adsorbent. The ideal adsorption parameters were 20 °C, 0.1 g of adsorbent dosage, pH of 1.0, initial Cr(VI) concentration of 15 ppm, and 180 min of contact time. The pseudo-second order kinetic model showed the best explanation of the adsorption process, based on the kinetic studies. The Temkin model gave the most accurate description of the adsorption process among the applicable adsorption isotherm models. According to the thermodynamic studies, Cr(VI) adsorption on SDS-BC was an exothermic, spontaneous process. Although this study demonstrates the effectiveness of SDS-BC in uptaking Cr(VI) ions, one should state that adsorption experiments were conducted under controlled laboratory conditions, which may not fully replicate the complexity of real wastewater systems. Additionally, the long-term stability of the modified biochar requires further evaluation to ensure its practical viability. Future research should focus on scaling up the production process, testing performance under diverse environmental conditions, and exploring cost-effective regeneration methods to enhance its sustainability.

## Data Availability

No datasets were generated or analysed during the current study.
